# Bovine Leukemia Virus: Origin, Prevalence, Phylogenetic Diversity, Risk Factors, and Strategies for Control

**DOI:** 10.3390/ani15091344

**Published:** 2025-05-07

**Authors:** Yuxi Zhao, Jiandong Wang, Jianguo Chen, Yingyu Chen, Changmin Hu, Xi Chen, Aizhen Guo

**Affiliations:** 1 National Key Laboratory of Agricultural Microbiology, Hubei Hongshan Laboratory, College of Veterinary Medicine, The Cooperative Innovation Centre for Sustainable Pig Production, Huazhong Agricultural University, Wuhan 430070, China; zyxwpc18214@gmail.com (Y.Z.); chenjg@mail.hzau.edu.cn (J.C.); chenyingyu@mail.hzau.edu.cn (Y.C.); hcm@mail.hzau.edu.cn (C.H.); chenxi@mail.hzau.edu.cn (X.C.); 2 Key Laboratory of Development of Veterinary Diagnostic Products, Key Laboratory of Ruminant Bio-Products, China Ministry of Agriculture and Rural Affairs, Huazhong Agricultural University, Wuhan 430070, China; 3 Institute of Animal Science, Ningxia Academy of Agricultural and Forestry Sciences, Yinchuan 750002, China

**Keywords:** bovine leukemia virus, origin, diversity, prevalence, risk factors, control

## Abstract

Bovine leukemia virus (BLV) is classified as a *Deltaretrovirus* and shows close genomic and biological similarities with human T-cell leukemia viruses (HTLVs). It serves as the etiological agent for enzootic bovine leukosis (EBL), which stands as the most prevalent neoplastic disease affecting cattle globally. Additionally, BLV has been identified as a potential zoonotic pathogen, although the risk to human health remains a subject of ongoing research. The insidious nature of BLV lies in its predominantly subclinical presentation; the majority of BLV-infected cattle show no apparent symptoms. This subclinical nature poses challenges for disease detection and control, as infected animals can remain unnoticed carriers, contributing to the silent spread of the virus within herds. This characteristic also underscores the importance of surveillance and early detection strategies to monitor BLV prevalence and mitigate its spread. Despite concerted efforts in some regions to implement eradication programs, BLV continues to maintain a high prevalence worldwide. The persistence of BLV in cattle populations highlights the need for innovative and integrated approaches to control and manage the disease effectively. The development of a BLV vaccine represents a significant breakthrough in the fight against BLV transmission. A successful vaccine can not only reduce the incidence of BLV infection but also minimize the associated economic losses linked to reduced milk production, reproductive issues, and the premature culling of infected animals. Therefore, a comprehensive understanding of BLV, encompassing its origin, evolutionary patterns, epidemiology, risk factors, and control strategies, is pivotal. Such knowledge serves as the foundation for the development of effective vaccines, diagnostic tools, and control measures. Through this review, we aim to consolidate and present this multifaceted understanding of BLV, providing valuable insights and guidance for researchers, veterinarians, and policymakers involved in BLV prevention and control efforts globally.

## Simple Summary

Bovine leukemia virus (BLV) is a virus that causes enzootic bovine leukosis, a common cancerous disease in cattle worldwide. Although most infected cattle are asymptomatic, they can still spread the virus, making detection and control challenging. There are ongoing concerns regarding the potential risk of BLV to human health. Despite efforts to eliminate the virus in some regions, it remains widespread, underlining the need for innovative strategies to manage the disease. A vaccine for BLV could be a significant breakthrough, reducing infections and mitigating economic losses associated with milk production, reproductive issues, and premature culling of infected animals. Understanding BLV’s origins, transmission routes, and risk factors is crucial for developing improved prevention, diagnostic tools, and control measures. This review consolidates current knowledge on BLV, aiming to inform researchers, veterinarians, and policymakers to enhance global efforts in combating the disease.

## 1. Introduction

Bovine leukemia virus (BLV) is classified within the genus *Deltaretrovirus* in the family Retroviridae. This genus additionally encompasses human T-lymphotropic virus types one, two, three, and four (HTLV-1, 2, 3, and 4) and simian T-cell leukemia virus types one, two, and three (STLV-1, STLV-2, and STLV-3) [1]. BLV is the causative pathogen of enzootic bovine leukosis (EBL), a widespread hematogenous neoplastic disease in cattle with a global distribution.

BLV is a single-stranded, diploid RNA virus with a complete genome comprising an 8714-nucleotide sequence. The BLV genome exhibits a conservative and modular structure, with a suite of structural genes (*gag*, *pro*, *pol*, and *env*) at the 5′ end of the genome, followed by auxiliary genes (*G4*, *R3*, and microRNA) and regulatory genes (*tax* and *rex*) at the 3′ end. Two identical long terminal repeat (LTR) sequences are located at both the 5′ and 3′ ends of the genome [2]. The viral infection cycle is initiated by viral particles, which mediate host cell entry through attachment, reverse transcription, and integration of the proviral DNA into the host genome [3]. Once integrated, the BLV genome persists as proviral DNA, maintaining a largely conserved and intact form with minimal deletions observed in infected cattle [4]. The virus predominantly disseminates through the transfer of infected lymphocytes into susceptible hosts [5].

Approximately 70% of cattle infected with BLV exhibit subclinical infections, which are asymptomatic and can easily be overlooked. This makes it difficult to identify infected animals early, contributing to a high prevalence of BLV in cattle herds [6]. Although diagnostic tests for BLV are available, subclinical infections often remain undetected without routine surveillance, making it challenging to effectively control the virus. Approximately 30% of BLV-infected cattle develop persistent lymphocytosis (PL), a chronic condition that typically does not exhibit the severe clinical signs associated with malignant B-cell lymphoma. However, between 1 and 5% of BLV-infected cattle may develop malignant B-cell lymphoma after a prolonged incubation period, leading to more severe clinical symptoms such as anorexia, dyspepsia, decreased milk production, persistent bloat, abomasal displacement, diarrhea, constipation, enlarged superficial lymph nodes, lameness, paralysis, weight loss, generalized weakness or debility, and occasionally neurological signs [7]. These symptoms are primarily linked to malignant B-cell lymphoma, not to persistent lymphocytosis.

Furthermore, it has recently been hypothesized that BLV infection diminishes the energetic efficiency of cows by altering the function of their rumen and intestinal microbiota, a process partly dependent on the proliferative capacity of BLV strains [8]. Additionally, BLV induces dysfunction in monocytes and neutrophils, ultimately leading to immunosuppression. These effects may increase the susceptibility of animals to secondary infections, compromise milk production, and reduce fertility [9]. The subclinical nature of the infection, the challenge of detecting infected animals early, and the difficulties related to routine surveillance are key factors contributing to the impracticality of controlling the disease.

The purpose of this review is to elucidate the origin, phylogenetic diversity, epidemiology, risk factors, and prevention and control strategies of BLV, aiming to provide comprehensive insights into the virus for researchers, veterinarians, and policymakers.

## 2. Origin and Diversity of BLV

### 2.1. Origin of BLV

Researchers used BEAST v 2.4.8 software to investigate and infer the origin of BLV and proposed that BLV originated in Asia. The estimated time to the most recent common ancestor (tMRCA) of BLV is suggested to date back to 1761 (95% highest posterior density: 1588–1871), with Bos taurus indicus indigenous to the region identified as the initial host. Subsequently, the virus is thought to have primarily propagated to other world regions through the international cattle trade, while the domestication and diversification of various Asian cattle breeds, including yaks and water buffaloes, are believed to have played a role in the emergence and sustained presence of BLV [10].

### 2.2. Evolutionary Driving Force

#### 2.2.1. Adaptive Drive

Comparative genomic analysis demonstrates that BLV and HTLVs possess strong homology in the *gag* and *pol* genes, suggesting a close evolutionary relationship between the two. However, the surface glycoprotein segment of the *env* gene exhibits low homology, indicative of differences in host range. This low glycoprotein homology implies that BLV and HTLVs may mediate cellular invasion by binding to disparate receptors on the host cell surface, thus adapting to varied host environments through evolution [11]. BLV displays substantial genetic diversity, with multiple genotypes identified across differing geographical regions. The distribution of BLV genotypes in various regions around the world illustrates the influence of geographic distance on virus evolution [12]. Considering the fact that BLV diversity results from mutations, particularly in the *env* genes, these mutations are also hypothesized to arise under functional constraints, highlighting the fact that BLV evolution is shaped by the necessity to adapt or preserve specific biological functions [13].

#### 2.2.2. Coevolution and Interaction

Host–pathogen coevolution is a complex process, potentially leading to increased susceptibility and genetic variation in response to infection. The outcome of the interaction between host and pathogen is contingent upon their coevolutionary dynamics. Studies have identified mutations at the N-linked glycosylation site of a single-envelope protein in BLV, resulting in enhanced fusion capabilities and protein stability [14]. This mutation not only ensures the efficient replication and transmission of BLV but also avoids inducing excessive pathogenicity that could compromise its persistence. Specifically, this refers to the development of lymphoproliferative diseases in animals following a prolonged incubation period. The balance between the virus and its host can be disrupted by the emergence of more pathogenic strains, suggesting a coevolutionary mechanism between BLV and its host [3]. Furthermore, BLV infection can impact both the innate and adaptive immune systems and also alter the function of uninfected cells. Such changes can result in abnormal immune responses, potentially promoting the persistence and propagation of the virus within the host population [15].

#### 2.2.3. Immune Evasion

BLV utilizes genetic mutations to evade immune system recognition. For example, natural mutations in the BLV Tax protein and LTR region can alter its function, leading to a decrease in the transcriptional activity of the LTR promoter, potentially having a significant impact on the proviral load (PVL), viral adaptability, and transmission ability in BLV-infected cattle [16]. Mutations in the *env* gene can result in antigen variants that evade immune testing. Research indicates that amino acid substitution at the G and H epitopes of the *env* gene plays a crucial role in virus escape strategies [17]. These mutations may impact the infectivity of the virus and its ability to be recognized by the host immune system, thus facilitating its persistence and spread within the host’s body [18]. Additionally, BLV is capable of disrupting immune responses through a single nonsynonymous mutation in CD4^+^T cell epitopes. These mutations diminish the binding strength of epitopes to bovine leukocyte antigen-DR (BoLA-DR) molecules (the bovine equivalents of class II molecules in human major histocompatibility complexes), resulting in a weakened immune response [19]. In a study, a detailed molecular analysis was performed on the complete BLV genome isolated from bovine B lymphosarcoma samples. Nucleotide substitution occurred in the glucocorticoid-responsive element site of BLV isolate 5′LTR. This mutation can potentially enhance the virus’s ability to evade the immune system by altering its expression in malignant B cells and might serve as a strategy to evade effective immune attacks [20].

These mechanisms facilitate BLV persistence in the host’s body, leading to chronic infection and disease progression, thus enabling its survival and reproduction within the host population, and constitute the primary driving force behind BLV evolution.

### 2.3. Global Phylogenetic Diversity

The global genetic diversity of BLV constitutes a significant research focus, particularly regarding the *env* gene that encodes the viral envelope glycoprotein, critical for the virus’s infectivity and antigenicity [18]. The BLV genotype has been determined through phylogenetic analysis of both the whole genome and the *env* genes. To date, BLV sequences have been classified into 12 different genotypes [12,18,21]. The genetic diversity of BLV is significantly influenced by geographical distribution, with genotype 1 (G1) being the most common and widely distributed genotype globally [12]. G1 is distributed across almost all continents, including Europe, America, Asia, and Oceania. In South America, genotypes G1, G2, G3, G4, G5, G6, and G9 are prevalent [12,22]; in comparison, G4, G7, and G8 predominate in Russia, Mongolia, and Eastern Europe [12,23]. Genotypes G6 and G10 are predominantly found in China, Jordan, India, Myanmar, Thailand, Vietnam, and the Philippines [12,24]. Genotypes G1, G4, G6, G10, and G11 are prevalent in China [13,25]. Genotypes G11 and G12 are exclusively prevalent in China and Kazakhstan, respectively [21,25].

## 3. BLV Endemic

### 3.1. BLV Infection Host Spectrum

Although BLV is nominally specific to certain hosts, it exhibits a broad host range. The broad host range of BLV is attributable to its ability to bind with the receptor cationic amino acid transporter 1 on target cells, a receptor that is non-specific to any species and expressed in various cells across different species [26]. This receptor facilitates BLV entry into host cells. Reports indicate that BLV primarily infects cattle and buffalo; however, experimental transmission to several other animal species, including sheep, goats, pigs, rats, rabbits, and chickens, is possible. BLV can also be detected in human mammary epithelial cells [26].

Research on endogenous retroviruses, including BLV, indicates that these viruses have infected a wide range of mammalian species over a long evolutionary process. This suggests that the host range of BLV and related viruses might be broader than previously recognized [27]. In summary, BLV is not strictly limited to bovine species and might infect various mammalian hosts, including humans.

### 3.2. BLV Eradication Status

Since the 1960s, various countries in Europe have initiated efforts to control BLV. Owing to these proactive intervention measures, numerous countries including the UK, France, Germany, Spain, Belgium, Denmark, Sweden, Switzerland, and Poland, among others, have reported the official eradication of BLV [28]. In contrast, nations such as Italy and Portugal boast extensive BLV-negative regions, with infections confined to a limited geographical scope [12,28]. In Australia, the virus has been eradicated from dairy herds, and its prevalence among beef cattle remains low [28]. In Europe, control strategies encompass the detection, isolation, and culling of infected animals [12,29]. Such strategies are augmented with robust biosafety and management measures to reduce or eliminate BLV exposure, demonstrating the potential to apply pertinent measures to eradicate BLV. The success of these strategies is highly contingent upon reliable estimates of BLV prevalence [12,30].

### 3.3. Prevalence in Non-Human Animals

Approximately 150 years ago, EBL was first reported in Germany [30]. The causative pathogen, BLV, was first described in Lithuania in 1871 and isolated from infected cattle lymphocytes in 1969 [31]. With global trade intensification, BLV poses significant challenges to animal husbandry.

BLV varies across different regions and countries, including those with important trade relations with China. In China, BLV is notably prevalent, with infection rates reaching up to 49.1% among dairy cattle and 1.6% among beef cattle in animal level [32]. In the US, 94.2% of dairy cattle herds have at least one cow that is BLV-positive. The average within-herd prevalence of BLV antibodies in dairy cattle is approximately 46.5% [33]. In Japan, studies have reported a prevalence of 28.6% (2007) and 35.2% (2010–2011) at the animal level [34,35]. In addition, Canada saw an increase in prevalence from 88.39% in 2016 to 89.30% in 2018 in at the herd level [36]. In contrast, countries such as Australia and New Zealand, which also have important trade relations with China, have successfully eradicated BLV from dairy herds [28]. In contrast, Argentina, a major exporter of beef to China, is experiencing an alarmingly high prevalence of BLV, with individual-level prevalence reaching 77.4% and herd-level prevalence at 90.9% [12]. Russia, another key trade partner with China, faces major challenges with BLV, particularly in its Central Federal District, where there were 4.98-fold and 10.24-fold increases in cases in Belgorod and Bryansk from 2012 to 2016 [37].

In addition to these countries, BLV has also been identified in other countries. In Colombia, individual prevalence varied between 17.0% and 62%, with the population prevalence reaching as high as 92% [38,39]. BLV has also been detected in sheep and water buffalo in Colombia, with an individual prevalence of 34.1% and 19.7%, respectively [40]. Thailand has reported a varying individual prevalence, from 5.3% to 87.8% [41]. In Kazakhstan, the individual prevalence ranges from 0.8% to 84% [21]. In comparison, Mongolia [23], Vietnam [24], and Myanmar [42] have reported individual-level prevalences of 3.9%, 21.1%, and 9.1% to 37.04%, respectively. The varied prevalences across these regions underscore the importance of monitoring and controlling BLV, especially in countries with important trade relations with China.

### 3.4. Prevalence in Humans

Regarding human health, the prevalence of BLV associated with breast cancer exhibits variation according to differing scientific investigation methods and geographical locations. The results of a meta-analysis, which included breast cancer samples from the US, Colombia, Brazil, Australia, Iran, Pakistan, and Jordan, revealed that BLV was present in 36% of the samples [43]. In Australia, BLV was identified in 80% of breast cancer samples and 41% of control samples [44]. In the USA, 59% of breast epithelial samples from breast cancer patients were found to contain BLV DNA; in comparison, the proportion in the normal control group stood at 29% [45]. In a Brazilian study, the prevalence of BLV in breast cancer samples was reported to be 95.9% [46]. The results of another study indicated that BLV DNA is detectable in 16% of breast cancer tissue samples from Egyptian women [47]. However, it is notable that, although the authors of some studies have suggested that BLV may be implicated in the onset of breast cancer, additional research is necessary to confirm BLV’s role in human cancer development [43].

## 4. Risk Factors

### 4.1. Cattle Movement and Trade

Cattle movement and trade, both domestic and international, have been identified as significant risk factors for the transmission of BLV. Multiple studies have shown that the introduction of new cattle to a farm is positively associated with the likelihood of introducing BLV-infected animals [48]. The number of new animals and the prevalence of BLV in their herd of origin are key contributors to this risk [49]. Additionally, uninfected cattle living in close proximity with infected cattle have a significantly higher risk of seroconversion [50]. Phylogenomic studies have suggested that BLV likely originated in Asia and spread to other continents through the international trade of live cattle, highlighting the role of global cattle movement in virus dissemination [10].The impact of BLV on trade is evident in international regulations, with restrictions often placed on semen from BLV-seropositive bulls due to transmission risks [51]. These findings underscore the need for stringent control strategies in the cattle trade, improved management practices, and stricter regulations to reduce the risk of BLV transmission. Effective monitoring and control measures are essential to minimize these risks and prevent further spread of the virus both domestically and internationally.

### 4.2. Host Genetic Factors

Genetic factors are crucial in determining the susceptibility of animals to BLV infection [52]. A principal genetic factor associated with BLV infection involves the polymorphism of the bovine leukocyte antigen (BoLA) complex, in particular, the BoLA-DRB3 allele. Certain BoLA-DRB3 alleles correlate with a high proviral load (HPVL) of BLV, signifying increased susceptibility to the virus [53]. Conversely, certain BoLA-DRB3 alleles are linked to HPVL resistance, suggesting that these alleles could bestow a degree of resistance to BLV infection [54]. The authors of a study in Colombia identified the Holstein breed as a risk factor for BLV infection; in contrast, membership of the Norman and hybrid breeds served as protective agents [55].

### 4.3. Age and Parities

Age is a significant risk factor for BLV infection. BLV prevalence is notably higher in adult cattle, and the risk of infection increases with age [56]. This increase may primarily be attributed to a heightened risk of exposure to BLV over time, as older animals are more likely to have had multiple opportunities for exposure to infected animals or contaminated environments. A study found that the age bracket of 1–2 years acts as a protective factor against BLV infection, whereas both animals younger than 1 year and older than 4 years face an increased risk of exposure and infection [55].

Additionally, parity (the number of calves born to a cow) is associated with an increased likelihood of BLV infection. Studies have shown a strong positive correlation between higher parity and BLV seroprevalence [57]. This correlation is likely due to the increased exposure risk associated with repeated calving, which may lead to greater contact with contaminated environments or infected animals.

### 4.4. Vertical Transmission and Feeding

Vertical transmission entails the transmission of a virus from the mother to the offspring, potentially occurring in the uterus or during childbirth. Approximately 10% of calves born to cows infected with BLV present with infection at birth [58]. This finding implies that the placenta and umbilical cord blood could serve as pathways for the vertical transmission of BLV. Colostrum, the milk secreted by the mammary glands of mammals immediately post delivery, represents another potential source of BLV transmission. Consumption of infected colostrum can lead to postpartum vertical infections from mother animals to calves [59]. Furthermore, the intake of BLV-positive milk by calves has been identified as a risk factor for infection [60].

### 4.5. Management Factors

The equipment utilized in farm production processes, such as syringes, obstetric gloves, dehorning implements, branding forceps, ear label forceps, medication bottles, hoof knives, nose clips, and delivery bars, together with routine procedures such as rectal examination and natural mating, could establish a horizontal transmission route from infected to healthy animals through direct contact with blood and fluids containing blood components. This horizontal transmission route, due to its association with the transfer of bodily fluids, is considered a principal risk factor for BLV infection [61]. Furthermore, increased stocking densities might elevate the risk of BLV transmission [62]. Herd size constitutes an important risk factor, as herds numbering more than 200 heads exhibit a heightened risk of BLV infection. This phenomenon could be attributed to an augmented possibility of direct contact between infected and uninfected cattle, particularly during feeding and drinking, heightening the risk of horizontal transmission [57].

### 4.6. Insect Vector

The bites of certain insects, particularly those of large, blood-feeding vectors such as *Tabanid* spp., have been identified as potential vectors for the spread of BLV [63]. Research has indicated that BLV may be mechanically transmitted via insect mouthparts; however, while insect bites might facilitate BLV transmission, they do not constitute the primary risk factor [28,64].

The risk factors for BLV infection vary significantly by region, reflecting the complex interplay of environmental, management, and biological factors that influence the spread and control of the disease. Understanding these regional differences is crucial for developing effective local strategies to manage and potentially eradicate BLV.

## 5. BLV Prevention and Control Strategies

Strategies to control and prevent BLV infection encompass a range of methods, with a primary focus on detection and treatment, resistance breeding, vaccine research and development, vaccination, and the establishment of robust farm management regulations.

### 5.1. Test and Processing

The foundational strategy for BLV control involves identifying infected animals, followed by subsequent actions. This approach depends on detection methods, such as ELISA and PCR, to identify BLV antibodies or proviral DNA, respectively. The detection and elimination strategy, encompassing regular testing of cows for BLV and culling those that are infected, is currently among the most effective means of controlling BLV spread [29]. Several European countries have eradicated BLV by adopting this strategy and integrating biosafety measures, providing a model for BLV eradication and demonstrating the strategy’s effectiveness [28]. Although efficient and capable of achieving a BLV-free state swiftly, this approach is costly, particularly when initial BLV prevalence is high, necessitating continuous monitoring and the backing of official compensation policies for success. In countries without economic compensation policies, including the United States, Japan, Canada, and Argentina, the implementation of this approach has been abandoned [28,34,65].

Testing and management constitute an alternative approach to lower costs through the implementation of biosafety and management measures that minimize animal exposure to infectious sources, in combination with diagnostic testing. This strategy allows for the identification and management of infected animals, reducing the need for mandatory early culling and the elimination of BLV-positive cattle, which can lead to significant cost savings. By using diagnostic testing alongside management practices, this approach helps control BLV infection while keeping costs lower compared to strategies relying on culling alone [29].

In regions characterized by high BLV prevalence, selective culling based on PVL presents a promising alternative. This strategy specifically targets the most infectious animals—those with high PVL—thereby reducing virus transmission risk without the need for mass removal of all seropositive animals. Recent studies confirm that PVL-based selective culling substantially decreases both BLV prevalence and incidence within herds [66,67]. Notably, this approach has been particularly valuable in regions where comprehensive culling strategies are logistically and economically prohibitive. In Argentina, for example, selective PVL-based culling has been proposed and implemented successfully as a more viable and economically acceptable alternative to blanket culling, mitigating herd health and economic impacts while effectively controlling the disease [68].

Ultimately, by combining diagnostic testing and PVL-based selective culling, producers can significantly lessen the economic burden of BLV control while effectively mitigating its spread. This integrated, targeted approach is particularly advantageous in regions facing high initial prevalence rates and economic limitations, offering a practical solution where comprehensive eradication strategies remain challenging.

### 5.2. BLV Resistance Breeding

Selective breeding for resistance to BLV represents a promising area of research, considering that studies have demonstrated that cattle exhibit varying degrees of susceptibility to BLV. The quantum of DNA that BLV integrates into the cattle’s host genome, termed PVL, serves as a crucial indicator of disease progression and transmission risk [69,70].

The *BoLA DRB3* gene has been studied as a molecular marker for BLV resistance. Particular alleles of this gene have been identified with either increased resistance or greater susceptibility to BLV infection. For instance, alleles including *BoLA-DRB3009:02*, *DRB3014:01:01*, and *DRB3002:01* show correlations with reduced BLV infectivity and LPVL, suggesting genetic resistance to BLV [19]. Conversely, alleles including *BoLA-DRB3015:01* and DRB3*012:01 are linked with higher BLV infectivity and HPVL, denoting susceptibility [54]. Other genetic elements, such as the *BoLA DQA1* gene, while linked to BLV resistance, do not exhibit as strong of an association as the *DRB3* gene [71].

While identifying these genetic markers holds promise for selective breeding programs, this strategy is not yet fully evaluated or validated as a routine method of BLV control. Selective breeding is still in the research and experimental phase, and its broader implementation will require further validation. Moreover, it is crucial to balance selective breeding with the preservation of genetic diversity within cattle populations to mitigate potential adverse effects on other traits and overall herd health [72].

### 5.3. BLV Vaccines

The development of effective BLV vaccines faces significant challenges due to the nature of retroviruses. BLV can stably integrate into the host genome and undergo prolonged incubation, evading immune responses and making complete infection prevention difficult [73]. Safety concerns also arise, as some vaccination strategies may escalate infection risk or interfere with recovery [73]. Past efforts involving the use of synthetic peptides, inactivated viruses, cell lysates, viral subunits, recombinant vaccinia, and DNA vectors have fallen short, failing to achieve the necessary balanced activation of humoral and cell-mediated immunity for adequate protection [74]. A successful vaccine requires the sustained and harmonized activation of both antibody-mediated and cell-mediated immune responses. However, previous endeavors have failed to attain this delicate balance, leading to only partial protection at most [73].

However, advancements have been made with regard to attenuated BLV vaccines. The pBLV6073DX strain, derived from pBLV344 in Belgium, has demonstrated promising safety and efficacy in preventing wild-type BLV infection. It induces robust anti-BLV immunity, with low replication detectable only by using sensitive methods, and lacks HTLV-like sequences to mitigate recombination risks [74]. While these results are encouraging, concerns remain over potential vaccine safety issues such as recombination with endogenous viruses, increased toxicity, and antigen alteration. There is a need for large-scale controlled trials to conclusively assess these risks [73,74].

Though these developments are promising, it is important to note that vaccines are not yet available for BLV control. The pBLV6073DX vaccine represents a milestone in BLV vaccine development; however, further research and evaluation are required to ensure comprehensive safety and widespread availability in the future.

### 5.4. Farm Management Control Program

Effective farm and veterinary management are keys to reducing BLV prevalence. Such management practices include: timely BLV testing of new cattle and culling/isolating infected animals based on assessments; preventing blood contact between animals by using disposable equipment and disinfecting tools; using BLV-negative semen and artificial insemination instead of natural mating; eradicating mosquitoes in the area and quarantining calves born to infected cows; providing colostrum from BLV-negative cows and enhancing veterinary practices and training; and utilizing bloodless dehorning methods and adhering to rigorous hygiene standards. Monitoring, economic assessment, and strategic research collaboration are also crucial. Programs such as New York’s Cow Health Insurance, developed through collaboration, offer structured BLV prevention and control based on best practices [50,75,76].

The control of BLV on farms requires a comprehensive approach that takes into account both biological and economic factors [29]. While successful strategies for managing BLV have been implemented in regions like Western Europe, the feasibility and effectiveness of these methods can differ significantly depending on local economic conditions, regulatory environments, and the level of public awareness. For example, in countries such as France, coordinated efforts involving government support, infrastructure improvements, and subsidies have led to more effective control [29,77]. In contrast, regions in South America and parts of Asia face significant challenges due to limited resources, insufficient government funding, and less stringent regulations, which hinder the widespread adoption of effective control measures [10].

Reducing BLV prevalence on farms necessitates a multifaceted approach that includes the systematic detection and elimination of the virus, as well as the reinforcement of biosafety protocols. Experience in European countries underscores the pivotal role of stringent biosafety measures in mitigating the spread of BLV, together with the inherent economic implications of these methods [73]. In regions exhibiting high BLV prevalence, the adoption of innovative strategies, encompassing vaccination and management measures to minimize exposure, becomes particularly crucial. The effectiveness of such measures is contingent upon the regional economy’s feasibility and the adaptability of management practices to local contexts. Such a comprehensive strategy necessitates the collaborative engagement of all stakeholders to ensure alignment with best practices and the integration of new research findings into herd management agreements, thus mitigating the impact of BLV on animal health and farm productivity.

## 6. Conclusions and Future Prospects

Based on the findings of this review, it is clear that BLV control programs should be emphasized in countries where BLV poses a significant risk to animal health and farm productivity. In economically stronger countries with well-established dairy industries, particularly in Western Europe, the implementation of systematic testing, culling of infected animals, and stringent biosafety measures should be prioritized. These regions benefit from strong governmental support, financial subsidies, and the economic importance of dairy exports, which make these control strategies more cost-effective.

However, in countries with fewer resources or where dairy farming is less economically critical, such as some regions in South America and Asia, the adoption of BLV control programs may face significant barriers due to the high costs associated with extensive testing and culling programs. In these areas, a more feasible approach would be to focus on basic biosecurity measures, targeted diagnostic testing, and the gradual introduction of more advanced measures, such as vaccination, as financial and infrastructural resources allow. Tailoring control methods to the local economic realities and specific challenges of each region is essential for the successful implementation of BLV control programs.

To move BLV closer to a controlled status, it is imperative to continue investing in research. This includes the development of more sensitive and reliable diagnostic tools, the creation of effective and safe vaccines, and further exploration of genetic resistance markers in cattle. Collaboration between governments, academic institutions, and the private sector will be key in advancing research and ensuring the practical application of these innovations. Moreover, consistent post-diagnostic and post-vaccination surveillance will be necessary to monitor the effectiveness of control programs, make adjustments as needed, and ensure long-term sustainability.

In conclusion, the direction for the most cost-effective control program method should focus on a region-specific, integrated approach that combines diagnostic testing, biosafety practices, and future vaccination strategies, supported by ongoing research and collaboration across stakeholders. With a concerted global effort and a strategic approach tailored to local conditions, it is highly likely that significant progress can be made in controlling BLV, thus mitigating its impact on cattle farming worldwide.

## Data Availability

This study did not involve the creation or analysis of any new data.
